# Approach to the Patient With Raised Thyroid Hormones and Nonsuppressed TSH

**DOI:** 10.1210/clinem/dgad681

**Published:** 2023-11-21

**Authors:** Carla Moran, Nadia Schoenmakers, David Halsall, Susan Oddy, Greta Lyons, Sjoerd van den Berg, Mark Gurnell, Krishna Chatterjee

**Affiliations:** Endocrine Section, Beacon Hospital, Dublin, D18 AK68, Ireland; Endocrine Department, St. Vincent's University Hospital, Dublin, D04 T6F4, Ireland; School of Medicine, University College Dublin, Dublin, D04 V1W8, Ireland; Wellcome Trust-MRC Institute of Metabolic Science, University of Cambridge, Cambridge CB2 0QQ, UK; Department of Clinical Biochemistry, Addenbrooke's Hospital, Cambridge CB2 0QQ, UK; Department of Clinical Biochemistry, Addenbrooke's Hospital, Cambridge CB2 0QQ, UK; Wellcome Trust-MRC Institute of Metabolic Science, University of Cambridge, Cambridge CB2 0QQ, UK; Department of Clinical Chemistry, Erasmus Medical Center, 3015 GE Rotterdam, The Netherlands; Department of Internal Medicine, Erasmus Medical Center, 3015 GE Rotterdam, The Netherlands; Wellcome Trust-MRC Institute of Metabolic Science, University of Cambridge, Cambridge CB2 0QQ, UK; Wellcome Trust-MRC Institute of Metabolic Science, University of Cambridge, Cambridge CB2 0QQ, UK

**Keywords:** thyroid function tests, thyroid hormone action, assay interference

## Abstract

Measurement of free thyroid hormones (THs) and thyrotropin (TSH) using automated immunoassays is central to the diagnosis of thyroid dysfunction. Using illustrative cases, we describe a diagnostic approach to discordant thyroid function tests, focusing on entities causing elevated free thyroxine and/or free triiodothyronine measurements with nonsuppressed TSH levels. Different types of analytical interference (eg, abnormal thyroid hormone binding proteins, antibodies to iodothyronines or TSH, heterophile antibodies, biotin) or disorders (eg, resistance to thyroid hormone β or α, monocarboxylate transporter 8 or selenoprotein deficiency, TSH-secreting pituitary tumor) that can cause this biochemical pattern will be considered. We show that a structured approach, combining clinical assessment with additional laboratory investigations to exclude assay artifact, followed by genetic testing or specialized imaging, can establish a correct diagnosis, potentially preventing unnecessary investigation or inappropriate therapy.

Measurement of circulating free thyroid hormones (THs) (thyroxine, T4; triiodothyronine, T3) and thyrotropin (TSH) using immunoassays is an essential step when assessing thyroid status, and produces characteristic patterns of thyroid function tests (TFTs) that correlate with classical thyrotoxicosis (raised THs, suppressed TSH) and hypothyroidism (raised TSH, subnormal THs), or deviate to generate anomalous or discordant TFTs due to different underlying causes (1). Here, we describe our approach to the investigation of patients with different patterns of biochemical hyperthyroidism: isolated, elevated free T4 [FT4] [hyperthyroxinemia]; isolated, raised free T3 [FT3] [hypertriiodothyroninemia]; combined elevation of FT4 and FT3 with nonsuppressed TSH. We review different categories of assay interference (eg, due to abnormal TH binding proteins, hormone displacement from binding proteins, antihormone (iodothyronines, TSH) or anti-assay reagent antibodies, biotin) causing spuriously abnormal hormone measurements. We consider some physiological (eg, T4 replacement), pathological (eg, nonthyroidal or acute psychiatric illness) and drug treatment (eg, amiodarone) contexts that are associated with this biochemical pattern, with other entities, outside the scope of this review, being discussed elsewhere (2). We outline genetic or acquired conditions that are associated with genuine hyperthyroxinemia (eg, genetic or functional deficiency of deiodinase enzymes), hypertriiodothyroninemia (dyshormonogenesis, resistance to thyroid hormone α, monocarboxylate transporter 8 [MCT8] deficiency) or both raised FT4 and FT3 (Resistance to Thyroid Hormone β [RTHβ], TSH-secreting pituitary tumor).

To exclude assay interference, we describe additional, simple tests that can be undertaken in many laboratories, even in resource-limited settings, and also complex investigations that are best undertaken in specialist centers. We discuss molecular genetic tests used to diagnose heritable causes of biochemical hyperthyroidism and nonsuppressed TSH.

Prismatic clinical cases, exhibiting different patterns of nonsuppressed TSH and biochemical hyperthyroidism, have been used to illustrate our diagnostic approach, which combines clinical, biochemical, and (if appropriate) genetic and/or radiological investigation.

## Clinical Cases

### Case 1

A 19-year-old woman, with a constellation of symptoms of thyrotoxicosis (anxiety, palpitations, insomnia), was found to have abnormal thyroid function tests (TSH 1.8** **mU/L [RR 0.35-5.5], FT4 **24 **pmol/L [RR 6.3-14] or **1.86** ****ng/dL [RR 0.48-1.08]), with similar results when her thyroid function was retested on two further occasions. Her mother, investigated for fatigue, showed similarly abnormal thyroid function (TSH 3.5** **mU/L [RR 0.35-5.5], FT4 **24 **pmol/L [RR 6.3-14] or **1.86 **ng/dL [RR 0.48-1.08]). Her maternal grandfather (deceased) was known to have had a thyroid problem of undefined nature.

### Case 2

A diagnosis of hypothyroidism (TSH **9.7 **mU/L [RR 0.35-5.5]) in a 67-year-old man with known type 2 diabetes mellitus and cardiomyopathy prompted treatment with 100** **µg of T4 daily. On this dose, discordant TFTs (TSH **13.7 **mU/L [RR 0.35-5.5], FT4 **69** pmol/L [RR 10.5-21] or **5.36 **ng/dL [RR 0.81-1.63]), led to discontinuation of therapy. Subsequently, a rise in circulating TSH (**45.6 **mU/L), together with a strongly positive antithyroid peroxidase antibody measurement (>**1300 **IU/mL [RR 0-60]), prompted recommencement of T4 (100** **µg daily). Puzzlingly, TFTs after T4 was restarted remained discordant (TSH **22.1** mU/L [RR 0.35-5.5]; FT4 **61** pmol/L [RR 10.5-21] or **4.74 **ng/dL [RR 0.81-1.63]).

### Case 3

Neonatal screening in a female infant showed TSH >**100 **mU/L (RR <10), prompting commencement of thyroxine therapy. Six years later, her brother was also found to have a raised TSH (**104 **mU/L) after birth (day 10), but levothyroxine therapy was withheld because his circulating total T4 (TT4) (109 nmol/L [RR 55-135] or 8.46** **μg/dL [RR 4.27-10.48]) and thyroid isotope scan were normal. Subsequent serial measurements recorded a spontaneous, progressive fall in TSH that normalized by age 18 months (Table 1) and he developed normally. This prompted a trial of levothyroxine withdrawal in his sister (age 7 years), following which her TFTs (Table 1) and thyroid isotope scan were normal. Although clinically euthyroid with no goiter, maternal TSH was raised (**60** ****mU/L) with normal TT4 (121 nmol/L, 9.40** **μg/dL) and negative thyroid autoantibody (antithyroid peroxidase, thyroglobulin, TSH receptor) measurements (Table 1).

**Table 1.** Case vignette 3: Thyroid function test results in family with raised TSH levels

	Daughter		Son					Mother
Age	Birth	7 years	10 days	1 month	3 months	6 months	18 months	28 years
TSH mU/L	**>100**	1.2	**104**	**80**	**37**	**18**	2	**60**
RR	<10	0.4-4	<10	0.4-4.0	0.4-4.0	0.4-4.0	0.4-4.0	0.4-4.0
TT4 nmol/L	n/a	127	109	106	117	131	120	121
RR	—	55-135	55-135	55-135	55-135	55-135	55-135	55-135

Figures in bold denote abnormal values.

Abbreviations: RR, reference range; TSH, thyrotropin; TT4, total thyroxine.

### Case 4

Investigation of a 64-year-old man with mitral regurgitation, atrial fibrillation, and impaired left ventricular function showed elevated circulating free TH levels (FT4 **31** pmol/L [RR 9-20] or **2.40** ng/dL [RR 0.69-1.55]; FT3 **8.3** pmol/L [RR 3-7.5] **5.40** pg/mL [RR 1.95-4.88]), with nonsuppressed TSH concentration (1.2** **mU/L [RR 0.4-4.0]). Magnetic resonance imaging visualized a low attenuation abnormality in the pituitary gland, prompting commencement of somatostatin receptor ligand (SRL) therapy for a presumed TSH-secreting pituitary microadenoma. A fall in circulating THs following treatment with octreotide 50** **µg subcutaneously 3 times a day for 7 days (Table 2) prompted continuation of therapy with monthly administration of depot SRL (Lanreotide Autogel 90 mg intramuscularly). However, following five months of this treatment, his TFTs remained abnormal (Table 2).

**Table 2.** Case vignette 4: Thyroid function test results in case 4 at baseline, after treatment with octreotide, and after treatment with lanreotide

	Baseline	Octreotide 50 µg SC tid, day 4 of administration	Octreotide 50 µg SC tid, day 7 of administration	After 5 months treatment with Lanreotide Autogel 90 mg IM
TSH mU/L	1.9	1.0	1.7	1.4
RR	0.4-4.0	0.4-4.0	0.4-4.0	0.4-4.0
FT4 pmol/L	**35**	**25**	**27**	**32**
RR	9-20	9-20	9-20	9-20
FT3	**9.6**	6.8	7.4	**9.4**
RR	3-7.5	3-7.5	3-7.5	3-7.5

Figures in bold denote abnormal values.

Abbreviations: FT3, free triiodothyronine; FT4 free thyroxine; IM, intramuscularly; RR, reference range; SC, subcutaneously; tid, 3 times daily; TSH, thyrotropin.

## Approach to Diagnosis

Considering the clinical context is helpful when evaluating a patient with discordant thyroid function. For instance, objective abnormalities (eg, goiter, clinical signs of hyperthyroidism, increased uptake on thyroid isotope scan) can suggest that the observed biochemical hyperthyroidism is genuine. Sedation with chloral hydrate (often undertaken in children) prior to blood sampling is a recognized cause of transient, reversible hyperthyroxinemia (3). Potential confounding factors, including altered physiological states (eg, pregnancy), intercurrent illness or concurrent medication should be considered (also reviewed in detail in (2)).

### Amiodarone Therapy

Changes in TFTs are almost universal in amiodarone-treated patients, with 2% to 24% developing overt thyroid dysfunction (4). The drug inhibits TH biosynthesis and pituitary type 2 deiodinase activity, prompting an initial rise in circulating TSH (1 week after commencement), which typically normalizes by 12 weeks (5, 6). Subsequently, the drug blocks hepatic, type 1 deiodinase activity, inhibiting the conversion of T4 to T3 and breakdown of reverse T3, resulting in a TFT profile of raised/high normal T4, low/low-normal T3, and markedly elevated reverse T3. This pattern may be evident as early as 2 weeks after commencement of treatment and can persist after drug withdrawal (5), with the changes in TFTs being dose dependent. Due to inhibition of T4 to T3 conversion, patients with amiodarone-induced hypothyroidism may require T4 replacement in supraphysiologic dosage to normalize TSH and therefore exhibit concurrently raised FT4 and TSH levels (5, 7).

### Nonthyroidal Illness

Prolonged or severe critical illness is often associated with abnormal TFTs; typically, circulating FT3 and FT4 fall (usually to low-normal levels), reverse T3 rises, and TSH values are low or low-normal. The degree of excursion in hormones is related to illness severity (6). Recovery is heralded by a rise in TSH with subsequent normalization of T4 and T3 (8, 9). Notably, changes in TFTs are not restricted to critical illness, with a low FT3, raised TSH and FT4 pattern being recorded in patients undergoing elective abdominal surgery (10) and in the weeks following myocardial infarction (11).

### Acute Psychiatric States

True hypothyroidism or thyrotoxicosis can be associated with psychiatric abnormalities (12). Conversely, transient hormonal changes (typically elevated circulating FT4 with nonsuppressed TSH) are seen in patients (∼15%) admitted with acute psychiatric states (13, 14), with this pattern being short lived, reversible, and not associated with a particular psychiatric condition (12, 14).

## Causes of Artefactually Raised Thyroid Hormones

### Familial Dysalbuminemic Hyperthyroxinemia

This dominantly inherited condition, due to heterozygous variants (Arg218His, Arg218Ser, Arg218Pro, Arg222Ile) in circulating albumin which alter its binding affinity for TH, causes artefactual elevation of FT4 and FT3 measurements with nonsuppressed TSH levels in euthyroid individuals, raising the possibility of Familial Dysalbuminemic Hyperthyroxinemia (FDH) being misdiagnosed as RTHβ or a TSH-secreting pituitary tumor. The commonest albumin variant (Arg218His), with a prevalence ranging from 1 in 10 000 in Caucasians to 1 in 100 in Hispanic populations (15), causes measurement interference in most currently available free TH immunoassay methods (16). A different variant at this position in albumin (Arg218Pro) binds steroid as well as iodothyronines, causing artefactual hyperthyroxinemia and hypercortisolemia (17). An albumin variant (Leu66Pro) with selectively increased affinity for T3, causing isolated hypertriiodothyroninemia, has been identified in a Thai kindred (18).

### Transthyretinemic Hyperthyroxinemia

Heterozygous variants (Gly26Ser, Ala129Thr, Ala129Val, Thr139Met) that increase the T4 binding affinity of transthyretin (TTR, also termed thyroxine-binding prealbumin), a protein that forms circulating, iodothyronine-binding tetramers, can also cause raised FT4 (and sometimes FT3) measurements in several, current immunoassay platforms (Table 3) (19), thus mimicking pathological conditions in euthyroid individuals. Confusingly, the occurrence of hyperthyroxinemia in genetically confirmed cases can be intermittent, with such variability being attributed to nonthyroidal illness or other factors affecting formation of heterotetramers by mutant TTR (15).

**Table 3.** Representative examples of entities which cause interference in free thyroid hormone measurements in different immunoassay platforms^a^

ENTITY*^b^*		Perkin–Elmer DELFIA	Beckman ACCESS/DXi	Abbott ALINITY	Roche COBAS	Siemens ATELLICA	Siemens ADVIA Centaur	Ortho VITROS	Fujirebio LUMIPULSE
*Transthyretinemic* *Hyperthyroxinemia* *T139M TTR mutation*	*TSH* *RR mU/L*	*2.19* *0.40-4.0*	*2.28* *0.34-5.6*	*1.96* *0.35-4.94*	*4.10* *0.27-4.20*	*2.35* *0.35-5.5*	2.20 *0.35-5.5*	2.34 *0.50-4.88*	2.1 *0.56-4.27*
	*FT4* *RR pmol/L*	*18.1* *9.0-20.0*	***21.0*** *7.7-15.1*	*17.0* *7.5-21.1*	***28.5*** *12.0-22.0*	***25.1*** *10.0-19.8*	**23.0** *10.5-21.0*	**25.8** *9.8-18.9*	**31.3** *13.5-24.3*
	*FT3* *RR pmol/L*	*NA*	*4.5* *4.3-6.8*	***3.3*** *3.8-6.0*	***7.6*** *3.1-6.8*	*5.3* *3.5-6.50*	4.9 *3.5-6.5*	6.1 *2.9-6.8*	5.1 *3.2-6.7*
	*TT4^c^* *RR nmol/L*	***167.0*** *69.0-141.0*							
*TBG deficiency* *F391S TBG mutation*	*TSH* *RR mU/L*	*1.03* *0.40-4.0*	*1.10* *0.34-5.6*	*0.98* *0.35-4.94*	*1.19* *0.27-4.20*	*1.14* *0.35-5.5*	1.13 *0.35-5.5*	1.18 *0.50-4.88*	1.00 *0.56-4.27*
	*FT4* *RR pmol/L*	***28.0*** *9.0-20.0*	*13.6* *7.7-15.1*	*10.5* *7.5-21.1*	*19.3* *12.0-22.0*	***29.3*** *10.0-19.8*	20.7 *10.5-21.0*	**21.5** *9.8-18.9*	18.2 *13.5-24.3*
	*FT3* *RR pmol/L*	*NA*	*3.8* *4.3-6.8*	*2.9* *3.8-6.0*	*3.5* *3.1-6.8*	*4.52* *3.5-6.5*	4.2 *3.5-6.5*	6.6 *2.9-6.8*	3.9 *2.7-6.0*
	*TT4* *RR nmol/L*	***44.4*** *69.0-141.0*							
	*TBG* *RR*	***<3.5*** *14.0-31.0*							
*Familial Dysalbuminemic* *Hyperthyroxinemia* *R218H ALB mutation*	*TSH* *RR mU/L*	*2.06* *0.40-4.00*	*2.12* *0.34-5.6*	*1.75* *0.35-4.94*	*2.09* *0.27-4.20*	*2.14* *0.35-5.5*	2.04 *0.35-5.5*	2.03 *0.50-4.88*	1.91 *0.56-4.27*
	*FT4* *RR pmol/L*	***20.8*** *9.0-20.0*	***39.8*** *7.7-15.1*	*18.4* *7.5-21.1*	***31.7*** *12.0-22.0*	***32.2*** *10.0-19.8*	**31.9** *10.5-21.0*	10.8 *9.8-18.9*	**31.3** *13.5-24.3*
	*FT3* *RR pmol/L*	*NA*	*6.8* *4.3-6.8*	***6.3*** *3.8-6.0*	***7.7*** *3.1-6.8*	***8.46*** *3.5-6.50*	**8.6** *3.5-6.5*	**6.9** *2.9-6.8*	**7.1** *3.2-6.7*
	*TT4* *RR nmol/L*	***248.5*** *69.0-141.0*							
*Anti-T4* *antibody*	*TSH* *RR mU/L*	***5.87*** *0.40-4.0*	***5.75*** *0.34-5.6*	***5.20*** *0.35-4.94*	***6.25*** *0.27-4.20*	***6.10*** *0.35-5.5*	**5.97** *0.35-5.5*	**6.2** *0.50-4.88*	**5.59** *0.56-4.27*
	*FT4* *RR pmol/L*	*14.7* *9.0-20.0*	*15.0* *7.7-15.1*	*15.8* *7.5-21.1*	***35.0*** *12.0-22.0*	***100.7*** *10.0-19.8*	**90.8** *10.5-21.0*	**37.2** *9.8-18.9*	**47.8** *13.5-24.3*
	*FT3* *RR pmol/L*	*NA*	*5.2* *4.3-6.8*	*5.9* *3.8-6.0*	*5.7* *3.1-6.8*	***2.95*** *3.5-6.5*	**3.4** *3.5-6.5*	5.5 *2.9-6.8*	5.7 *2.7-6.0*
	*TT4* *RR nmol/L*	***347.0*** *69.0-141.0*							

FT3 measurement using the DELFIA method is no longer available. Values for FT4, FT3 and TT4 are provided in SI units. Equations to convert to non SI units are; FT4 (pmol/L)×0.077688 = ng/dL; FT3 (pmol/L)/1.536 = pg/mL, TT4 (nmol/L)/12.87 = μg/dL.

Figures in bold denote abnormal values.

Abbreviations: NA, not available; RR, reference range; TBG, thyroxine-binding globulin; TT4, total T4.

^*a*^Two-step immunoassay methods are italicized, with other methods being one-step.

^*b*^For each entity, hormone measurements in the same sample from a case were undertaken in different immunoassays.

^*c*^Total T4 was measured by the DELFIA immunoassay method.

### Thyroxine Binding Globulin Deficiency

As expected, complete or partial deficiency of thyroxine binding globulin (TBG), due to defects in its gene on the X-chromosome, causes low circulating TT4 concentrations in affected males (15). However, although concurrent FT4 measurements are usually normal in these euthyroid individuals, this entity can also cause spuriously raised FT4 measurements in some immunoassay methods (Table 3) (20).

### Antibody-Mediated Interference

Categorization of antibody mediated assay interference is useful in designing strategies to overcome this rare but clinically significant issue. Interfering antibodies can either be directed against the hormone to be measured (autoantibodies), or to reagents within an immunoassay (antireagent antibodies). Antireagent antibodies can be further classified into either specific/high affinity or polyvalent/low affinity (heterophile) types (21). Due to their polyvalent nature, heterophile antibodies can cross link both the capture and detection antibodies in a 2-site immunoassay, causing a false positive signal in the absence of analyte. Rheumatoid factor, which can be classed as a type of autoimmune heterophile, can cause interference in immunoassays, including for THs or TSH (22, 23). Antireagent antibodies (which can be specific or heterophile) typically bind to the antibodies that either capture or detect the analyte in an immunoassay, but can also be directed against other assay elements (eg, the analogue tracer, linkers joining assay components). Current immunoassay designs are increasingly sophisticated, containing nonspecific Ig to adsorb anti-Ig antibodies or Fab fragment or chimeric antibodies (either lacking or with a humanized Fc portion), which reduce, but do not eliminate, this type of interference. Conversely, the increasing therapeutic use of monoclonal antibodies, particularly those which are not fully humanized, has the potential to increase the incidence of antibody interference.

### Anti-Iodothyronine Autoantibodies

Antibodies directed at iodothyronines (anti-T4 and anti-T3 autoantibodies), occurring at low prevalence (1.8%) in the general population but more frequently in patients with thyroid autoimmunity (especially hypothyroidism), can also bind labeled analogues of T4 or T3 used in immunoassays, leading to falsely elevated estimation of hormone values (24). Typically, assays with a two-step architecture, preventing contact between patient's serum and labeled analogue, are not susceptible to autoantibody-mediated interference, whereas one-step measurement methods are affected (25).

### Antireagent (Ruthenium) Antibodies

Heterophile antibodies directed against ruthenium, a tracer used by one manufacturer in electrochemiluminescence immunoassays, are a relatively common (up to 0.24%) (26) cause of falsely raised FT4 and FT3 or falsely low TSH measurements in assays of this type (24). The addition of blocking agents to ruthenium-based assays has greatly reduced, but not eliminated, this class of interference (26).

### Paraprotein-Associated Iodothyronine Assay Interference

Spuriously elevated total T3 but normal TSH and FT4 measurements, due to IgG paraprotein–mediated assay interference, has been recorded in cases of myeloma (27).

### Interference With Assay Architectures

The high affinity of interaction between biotin and streptavidin has prompted manufacturers to use this molecular link as part of the architecture of some, or all, of their immunoassays. While intake of 50μg of this B vitamin daily is sufficient to meet nutritional requirements, higher doses (5 to 30 mg) are used in neonates with suspected inborn errors of metabolism and in adults with multiple sclerosis. It is also widely marketed as a health supplement. Biotin ingestion in such high dosage causes interference with measurement of multiple thyroid (28) and nonthyroid analytes, with previous Roche, Beckman and Siemens Immulite immunoassays reported to be susceptible (29). Falsely elevated THs, with spuriously suppressed TSH and positive anti-TSH receptor antibody test results can mimic Graves disease (29). To avoid measurement interference, a recent study recommended withholding biotin intake for 24 hours prior to TSH, FT4, and FT3 measurement, or for longer to measure thyroglobulin reliably (30). The biotin–streptavidin link is also a target for antibody interference, and antibodies directed against streptavidin which interfere with susceptible TH and TSH assays have been described (31).

### Displacement From Binding Proteins

Heparin (polymeric or fractionated, low molecular weight), when administered intravenously or by subcutaneous injection, can activate endothelial lipoprotein lipase *in vivo*, releasing free fatty acids from circulating triglyceride, with such free fatty acids displacing free THs from binding proteins *in vitro*. This artifact is most evident when a blood sample is not processed for TH measurement for some time after being taken, or with laboratory assays requiring long incubation periods, and in patients with hypertriglyceridemia or hypoalbuminemia (32, 33). Blood sampling more than 10 hours after heparin administration and/or laboratory analysis without delay can minimize the risk of spurious free hyperthyroxinemia. In addition, concurrent normal TT4 values in the same sample are indicative of a displacement effect and can confirm the patient's euthyroid status. Administration of furosemide in high dosage (250-500 mg, orally or intravenously) is known to inhibit interaction of THs with its binding proteins, increasing measurements of FT4 (34), with even modern FT4 assays being variably susceptible to such interference (35). In such cases, TSH measurement may assess thyroid status more reliably.

## Causes of Spuriously Abnormal TSH

### Macro-TSH

Macro-TSH, an autoimmune, TSH–Ig complex of larger molecular size than TSH itself (36), is detected to varying extents by different TSH immunoassays, accounting for its variable (0.6-1.6%) estimated prevalence (24). Due to its high molecular mass, the half-life of circulating macro-TSH is prolonged; however, as this species is also restricted to the intravascular compartment, it is thought to be biologically inactive.

### Heterophile Anti-TSH Antibodies

As two-site immunometric assays are used almost universally to detect circulating TSH in clinical practice, they are susceptible to heterophile antibody interference (reported prevalence 0.4%) (37), which typically causes falsely high, or (less commonly) falsely low TSH measurements, depending on whether the antibodies are crosslinking or blocking. In practice, determining whether a spuriously raised TSH is due to interference from a crosslinking heterophile antibody or a macro TSH–Ig complex can be difficult, although once such assay interference is detected, the need to make this distinction is less clinically relevant.

### Rare Amino Acid Change (Arg75Gly) in TSHβ Subunit

A single nucleotide change in *TSHB*, encodes a variant TSHβ subunit polypeptide containing an amino acid change (Arg75Gly) that does not alter TSH function, but prevents recognition of TSH by monoclonal antibodies used in some immunoassays. The Arg75Gly TSHβ variant is particularly prevalent (1%) in South Asian populations, with heterozygotes and homozygotes exhibiting falsely low or even suppressed TSH measurements in some assay platforms (eg, Siemens ADVIA Centaur, IMMULITE 2000), sometimes resulting in misdiagnosis of hyperthyroidism (38), with other methods (eg, Roche Elecsys, Abbott ARCHITECT, or Beckman Coulter DxI) recording normal TSH (39, 40).

### Paraprotein-Associated TSH Assay Interference

Rare cases of falsely subnormal (Abbott AxSYM assay) or raised (Beckman-Coulter DxC 880i assay) TSH measurements due to interference from circulating IgG paraproteins have been recorded (41, 42), with interference resolving upon disappearance of the monoclonal gammopathy.

## True Biochemical Hyperthyroidism With Nonsuppressed TSH

### Genuinely Raised (Free) T4

#### Thyroxine therapy, including with poor compliance

Variable compliance with T4 therapy in hypothyroidism can cause a biochemical pattern of normal or elevated FT4 and raised TSH concentrations (43). In this context, a seemingly appropriate increase in thyroxine dosage (perhaps to a supraphysiological level) can normalize TSH levels but be associated with concomitantly elevated circulating THs, raising the possibility of underlying RTHβ (43). Distinguishing this clinical situation from true coincidence of RTHβ with autoimmune hypothyroidism (which occurs rarely), is discussed further below.

Separate to this, it is also recognized that a subset of hypothyroid patients, compliant with thyroxine therapy in physiological dosage, can exhibit raised circulating FT4 but normal FT3 and TSH concentrations (44, 45). This phenomenon has been attributed to diminished activity of type 2 deiodinase, reducing the generation of T3 (from its T4 precursor) that is available to inhibit pituitary TSH secretion (45).

#### Deficiency of selenocysteine-containing proteins, including deiodinase enzymes

Over 25 human proteins, including the thyroid deiodinase enzymes, contain the amino acid selenocysteine (Sec). The incorporation of Sec into selenoproteins during their translation is dependent on a unique cellular pathway that includes its own transfer RNA (Sec-tRNA^(Ser)Sec^, encoded by *TRU-TCA1-1*) and a protein (SECIS binding protein 2, encoded by *SECISBP2*), which binds a specific sequence (SElenium Cysteine Insertion Sequence [SECIS]) located in the 3′-untranslated region of all selenoprotein mRNAs. Biallelic mutations in *SECISBP2* cause a rare multisystem disorder, often presenting in childhood with growth retardation and developmental delay. Diminished activity of deiodinase enzymes and low circulating selenoproteins cause a distinctive biochemical signature of raised FT4, normal or low FT3, normal or raised TSH, elevated reverse T3 and low circulating selenium concentrations in all patients (Table 4); this biochemical pattern has also been documented in 2 patients with selenoprotein deficiency due to a homozygous nucleotide substitution in *TRU-TCA1-1* (46).

**Table 4.** Causes of isolated raised (free) T4 and normal TSH

Disorder	Thyroxine replacement therapy	Nonthyroidal illness	Acute psychiatric states	Amiodarone therapy	Selenoprotein deficiency*^a^*
FT4	High	High	High	High	High
FT3	Normal	Normal or low	Can be normal	Normal	Normal or low
TSH	Normal or raised	Normal	Normal	Normal	Normal
Reverse T3	Normal	Raised	Not known	Very Raised	Raised
Clinical context	Some individuals with acquired or congenital hypothyroidism	Sepsis or other clinical states	Acute admissions with psychosis or mood disorders	Raised FT4 can be an effect of amiodarone therapy, with persistent, isolated elevation not indicative of thyroid disease	Growth retardation Muscle weakness

Abbreviations: FT3, free triiodothyronine; FT4 free thyroxine; TSH, thyrotropin.

^*a*^Low plasma selenium levels are characteristic of selenoprotein deficiency due to mutations in *SECISBP2* or *TRU-TCA1-1*.

#### Loss of deiodinase enzyme function

In a family with dyshormonogenic congenital hypothyroidism (CH), heterozygosity for an additional mutation (Arg132His) in the type 1 deiodinase was associated with hyperthyroxinemia and raised reverse T3 concentrations, irrespective of whether or not they were on thyroxine replacement (47). Similarly, a combination of homozygosity for a common variant (Thr92Ala) in the type 2 deiodinase gene, together with heterozygosity for loss-of-function *TSHR* variants in two different hypothyroid patients, has been associated with raised circulating TSH and FT4 but normal total T3 in one individual and normalization of TSH only after addition of liothyronine therapy in the other patient, suggesting impaired conversion of T4 to T3 in both cases (48).

### Genuinely Raised (Free) T3 and T3/T4 Ratio With Nonsuppressed TSH

Some acquired or genetic conditions can be associated with high-normal/high circulating FT3 and low-normal/low FT4 concentrations, computing to a raised T3/T4 ratio, with normal TSH levels.

#### Dyshormonogenesis

Iodine deficiency, typically caused by a restrictive diet with inadequate intake of this element, can result in low circulating FT4 with slightly increased FT3 (due to preferential production of T3 over T4) and normal TSH concentrations, with chronic deficiency causing goiter formation (49). Progressive goiter, normal TSH, and raised circulating FT3 or an elevated T3/T4 ratio has been reported in the context of mild, dyshormonogenic CH due to partial, loss of function mutations in thyroid peroxidase (50) or thyroglobulin gene defects, with hypersecretion of T3 being attributed to increased thyroidal activity of pituitary type 2 deiodinase (51, 52). Increased thyroidal deiodinase enzyme activity in cases of metastatic follicular (53) or poorly differentiated thyroid cancer (54) also mediates higher circulating FT3 concentrations and an elevated T3/T4 ratio.

#### Resistance to thyroid hormone α

Heterozygous mutations in the thyroid hormone receptor α gene cause a disorder characterized by features of hypothyroidism, but associated with normal circulating TSH, low-normal/low FT4, high-normal/high FT3, and a raised T3/T4 ratio (55).

#### Monocarboxylate transporter 8 deficiency

Mutations in an X-linked gene (*SLC16A2*), encoding a membrane protein (MCT8) required for transport of THs into the central nervous system, cause a disorder (Allan–Herndon Dudley syndrome) with psychomotor retardation, elevated circulating FT3, low or low-normal FT4, and normal TSH concentrations (56).

Table 5 illustrates how entities sharing this pattern of TFTs can be differentiated, using a combination of clinical features and key biochemical measurements (eg, urinary iodine, serum thyroglobulin, and reverse T3).

**Table 5.** Causes of raised (free) T3 and elevated T3/T4 ratio

Disorder	Iodine deficiency	Mild dyshormonogenic congenital hypothyroidism*^a^*	Follicular or poorly differentiated thyroid cancer	RTHα*^b^*	Monocarboxylate transporter 8 deficiency*^c^*
Occurrence	Commoner in some world regions	Not known	Rare case reports	∼1 in 20 000-40 000	1 in 70 000 males
FT4	Low-normal or Low	Low-normal or Low	Low-normal or Low	Low-normal or Low	Low-normal or Low
FT3	High-normal or High	High-normal or High	High-normal or High	High-normal or High	High-normal or High
T3/T4 ratio	High	High	High	High	High
TSH	Normal*^d^*	Normal*^d^*	Normal	Normal*^d^*	Normal*^d^*
Reverse T3	Low	Not known	Low	Low or Normal	Low
Thyroglobulin	Raised	Raised*^e^*	Raised	Normal	Normal
Urinary iodine	Low	Normal	Normal	Normal	Normal

Abbreviations: FT3, free triiodothyronine; FT4 free thyroxine; RTHα, Resistance to Thyroid Hormone alpha.

^*a*^Due to mutations in genes (eg, *TPO*, *TG*) mediating thyroid hormone biosynthesis.

^*b*^Due to mutations in *THRA*.

^*c*^Due to mutations in *SLC16A2*.

^*d*^TSH can be slightly raised in severe iodine deficiency, mild dyshormonogenic CH, RTHα or some cases of MCT8 deficiency.

^*e*^Thyroglobulin is not raised in dyshormonogenic CH due to defects in *TG*.

### Genuinely Raised (Free) T4 and (Free) T3 With Nonsuppressed TSH

After excluding confounding causes (eg, measurement interference, effects of concomitant comorbidities or drug therapies) outlined above, genuinely raised, circulating FT4 and T3 with nonsuppressed TSH concentrations are compatible with either a genetic (RTHβ) or acquired (TSH-secreting pituitary tumor or thyrotropinoma, TSHoma) condition. Over 900 families with RTHβ, a dominantly inherited disorder caused by different (∼230 known), heterozygous mutations in the thyroid hormone receptor β gene (*THRB*), have been recorded worldwide (57). Greater detection of TSHomas likely mediates their higher prevalence (2.8 per million) than previously recorded (58). The age and gender of patients, and magnitude or pattern of elevation in free THs or TSH concentrations does not distinguish between these disorders (59). Although, in principle, a combination of genetic testing (for RTHβ) and pituitary imaging (for TSHoma) should readily differentiate these entities, a number of issues can cause diagnostic difficulties, as highlighted below.

Possibly due to sooner ascertainment, a greater proportion (25-30%) of TSHomas now present as microadenomas, with some pituitary lesions not discernible using standard magnetic resonance imaging (60) and being associated with a paucity or even absence of hyperthyroid signs and symptoms (58, 59, 61). Conversely, clinical features of hyperthyroidism can also be present in RTHβ (62) and incidental abnormalities on pituitary imaging are found in a significant proportion (20%) of genetically-proven RTHβ cases (59, 60).

Although dynamic endocrine tests (rise in TSH in response to thyrotropin-releasing hormone; inhibition of TSH following T3 administration) can differentiate between RTHβ and TSHoma, these investigations have limitations. For instance, thyrotropin-releasing hormone is not available in many countries. Moreover, although one case series confirmed that the TSH response is normal or exaggerated in RTHβ but blunted in TSHoma, with the authors specifying a greater than 5-fold TSH increase being a cut-off that differentiates these entities (60), there may be a “gray zone” (3- to 5-fold TSH response to thyrotropin-releasing hormone) compatible with either diagnosis (Gurnell, unpublished observations). T3 administration is not advisable in elderly individuals or those with underlying cardiac disease or psychiatric disturbance; furthermore, although circulating TSH is higher in TSHoma than RTHβ cases following the T3 suppression test, there is an overlap in TSH values with no discrete cut-off that distinguishes between these entities (60).

Circulating levels of sex hormone–binding globulin (SHBG) are normal in RTHβ (signifying hepatic resistance to TH action), but elevated in patients with TSHoma with hepatic hyperthyroidism (63). However, due to inhibition of its synthesis, SHBG can be normal in patients with mixed GH/TSH-secreting pituitary tumors or TSHoma with coincident insulin resistance or in microadenoma cases (59); conversely, other factors (oral estrogen therapy, anorexia) can cause elevated SHBG in RTHβ (2). An elevated molar ratio of circulating pituitary glycoprotein α-subunit to TSH can signify TSHoma, but this biomarker can be “falsely” elevated in postmenopausal RTHβ cases or normal in patients with micro-TSHoma (60).

Although finding genuinely elevated, circulating THs with nonsuppressed TSH in relatives of an index case is suggestive of a heritable disorder such as RTHβ, an absence of affected family members does not exclude the diagnosis, because 15% of RTHβ cases can occur sporadically due to a *THRB* mutation arising *de novo* (64). In 10% to 15% of cases with clinical and biochemical features consistent with RTHβ, *THRB* sequencing shows no abnormality. Here, diagnostic possibilities include a defect involving an as yet undiscovered gene mediating TH action, or somatic mosaicism for a *THRB* mutation that is not expressed in DNA from peripheral blood mononuclear cells (65). A recent report suggests that next-generation rather than conventional (Sanger) sequencing of DNA from patient cells of different embryonic origin is more sensitive at detecting somatic mosaicism (66).

## Apparent Hypothalamic–Pituitary–Thyroid Axis Resistance

It is well recognized that in some thyroxine-treated patients with goitrous or dysgenetic CH, but without any additional defect in genes mediating TH action, circulating TSH remains raised despite FT4 concentrations being within the normal range, such that a higher dosage of T4 (with resultant elevated FT4 concentrations) is required to normalize TSH (67, 68). This phenomenon has been attributed to CH altering the “setpoint” of the hypothalamic–pituitary–thyroid (HPT) axis during development, perhaps epigenetically (68). Previously, such axis resistance has been shown to lessen with age (69), but a recent study documented later development of HPT axis resistance in CH cases with previously normal sensitivity to TH during infancy (70).

## Co-occurrence of Different Entities

### FDH and Autoimmune Thyroid Disease

When FDH and thyrotoxicosis coexist, a suppressed TSH is associated with FT4 concentrations that are disproportionately raised or discordant when measured using different methods. Conversely, dramatic elevation in FT4 measurements when TSH is normalized with T4 treatment can indicate coincidence of FDH and autoimmune hypothyroidism (71).

### TBG Deficiency and Graves Disease

Investigation of a male patient with low serum TSH and TT4, but raised FT4 concentrations and thyroid stimulating immunoglobulins, identified hemizygosity for a pathogenic mutation in the *TBG* (*SERPINA7*) gene, hence signifying coincident TBG deficiency and Graves disease (72).

### RTHβ and Thyroid Autoimmunity

Graves disease or thyroiditis, with concomitant underlying RTHβ, is suspected when antithyroid drug treatment of a patient with conventional features (thyrotoxic symptoms, raised THs, subnormal or suppressed TSH) results in a marked, exaggerated rise in TSH concentration in the face of normal, circulating THs (73). Similarly, having excluded variable compliance, malabsorption or accelerated metabolism of TH (43), a requirement for thyroxine replacement in supraphysiological dosage (above 1.6-1.8** µ**g/kg body weight) to normalize circulating TSH in autoimmune hypothyroidism, in conjunction with raised FT4 and FT3 concentrations, can suggest underlying, coexistent RTHβ (74).

### RTHβ and TSH-Secreting Pituitary Tumor

Although pituitary abnormalities in RTHβ patients are usually incidental (see above), genuine coincidence of TSH-secreting pituitary microadenoma with genetically-confirmed RTHβ was described in a 12-year-old child who had raised free THs but elevated TSH and blunted responses in a T3 suppression test (75).

### RTHβ and Congenital Hypothyroidism

As discussed above, whilst persistently raised TSH with hyperthyroxinemia due to an altered HPT axis setpoint is recognized in thyroxine-treated CH (68), this can rarely be due to coexistence of CH and RTHβ, with failure to normalize TSH despite marked elevation in both FT4 and FT3 concentrations, or a requirement for levothyroxine in very supraphysiologic dosage, being suggestive of a dual disorder (76, 77).

## Methods to Detect Analytical Interference in Assays or Diagnose Entities

The ability to detect assay interference, and to estimate the true concentration of circulating THs or TSH, will depend on additional methods available in the clinical laboratory. Some additional tests can be undertaken in a general laboratory, whereas other measurements are best performed in a specialist reference laboratory. As even the simple methods described here cannot be used on every sample, interpreting results in conjunction with clinical context (eg, discordance between TFTs and clinical status of patient) or knowledge of laboratory systems (eg, an unexpected change in TFT pattern compared with previous measurements) can be very helpful. Grossly discordant, widely variable, or extremely deranged results usually raise suspicion of interference; unhelpfully, it is more subtle interference, causing plausible biochemical patterns, which can lead to diagnostic confusion and possibly unnecessary further investigation or intervention (24). Furthermore, in some situations, the presence of assay interference precludes accurate estimation of true TSH or TH values, requiring management of patients using clinical thyroid status rather than laboratory measurements.

## Simple Methods

### Method Comparison

Comparing hormone measurements made using two (or more), judiciously chosen, comparator assay platforms can identify most forms of immunoassay interference. For instance, assays using differing capture antibody species or affinities, sample dilutions or interference blocking agents, can expose antireagent or anti-analyte antibody interference. “Two-step” free TH assays are not susceptible to anti-iodothyronine antibody mediated interference (25). Measurement of free THs using assays with differing buffer or incubation conditions can expose interference due to genetic variants of circulating TH binding proteins (16) (Table 3). Comparison with an assay architecture that does not use biotin can be used to detect biotin and streptavidin antibody interference. Current initiatives to harmonize immunoassays (78) may limit the ability to detect assay interference by exploiting differences between measurement methods, placing greater reliance on analytical techniques described below.

### Polyethylene Glycol Precipitation

This test relies on the concept that addition of polyethylene glycol (PEG) to a serum sample preferentially precipitates either immunoglobulin-bound analytes or an antireagent antibody and is widely used to detect these classes of interference (79). Whilst cost-effective and readily available in most clinical laboratories, this method is not infallible and test protocols need to be carefully controlled. In the absence of antibody interference, PEG can precipitate up to 70% of native TSH, equating to greater than 30% recovery of this analyte in the test; in contrast, in cases of significant TSH assay interference, PEG can precipitate almost all macro-TSH or antireagent antibody, equating to less than 5% recovery of native TSH. However, with this test, there is a gray zone (5-30% TSH recovery) which signifies the presence of assay interference, but whether the magnitude of such interference has clinical significance is uncertain. Furthermore, in cases of TSH assay interference, the variability of this test also makes it difficult to accurately estimate the true TSH value. Elimination of such interference by incubating serum with protein G or anti-Ig agarose can confirm the presence of an interfering immunoglobulin (79).

As free TH immunoassays are exquisitely sensitive to matrix effects, the use of a PEG precipitation test to detect antibody-mediated interference in such assays is not recommended; better methods for measurement of free hormones (eg, equilibrium dialysis or ultrafiltration) which can overcome this type of interference are available (see below).

### Dilution Studies

When serum containing native TSH is diluted serially, its measured concentration should fall proportionately; in contrast, the presence of antireagent antibody or macro-TSH usually (but not always) causes a nonlinear reduction in measured TSH following dilution. As with PEG precipitation, this test needs to be conducted carefully using an appropriate diluent to preserve the assay matrix. Similarly, a nonlinear fall in total TH measurements following serial dilution can signify interference due to antireagent or anti-iodothyronine antibody. In contrast, as free THs re-equilibrate with sample dilution, this test cannot be undertaken using free TH assays (80).

### Interference Blocking Agents

Most commercial immunoassays incorporate proprietary reagents to neutralize either heterophile or anti-animal antibodies. When serum is assayed in the native (undiluted) state, high concentrations of interfering antibody may render these blocking reagents ineffective, generating a false result. In contrast, when a sample is assayed in dilution, favoring removal of the diluted interfering antibody by blocking agent, loss of interference can generate an accurate result.

In addition, it is possible to “preabsorb” antibody-mediated interference using dedicated, commercially available, blocking tubes or reagents before a sample is immunoassayed. In practice, this maneuver is a helpful but relatively ineffective way of eliminating antibody interference, possibly because many immunoassays have already incorporated reagents to block such interference.

## More Complex Methods

### Direct Methods for Measurement of Free Thyroid Hormones (Equilibrium Dialysis/Ultrafiltration)

Direct methods, which require physical separation of THs from their binding proteins prior to measurement of their concentration (typically by mass spectrometry) are intrinsically more accurate than indirect immunoassay methods. Equilibrium dialysis and ultrafiltration methods, separating free and protein-bound hormone, are exquisitely sensitive to analytical conditions which need to be carefully controlled (81, 82). Consequently, these assays are best undertaken in reference, rather than routine, clinical laboratories, but remain methods of choice when interference in free TH immunoassays is suspected. However, in FDH, even FT4 measured by direct dialysis can be raised in rare cases (83). It should also be noted that interference due to displacement of free THs (eg, heparin), will also affect these methods.

### Gel Filtration

A gel filtration method can detect interfering species if they are of different size to the native analyte and is particularly useful in defining “macro hormone” (eg, hormone–Ig) complexes (36, 84). This method is labor intensive and best undertaken in a specialist laboratory. In addition to detecting the macro-hormone complex, this method can potentially measure the uncomplexed free hormone—knowledge that could aid patient management. Unfortunately, macro-TSH–Ig complexes are often weakly bound and can dissociate during gel filtration, often confounding estimation of the true, biologically active, TSH concentration (85).

### Radiolabeled Binding Studies

When assayed under specific conditions, the binding of radiolabeled T4 to serum can detect variant albumin proteins causing dysalbuminemic hyperthyroxinemia (86). A combination of radiolabeled T4 and serum electrophoresis can detect abnormal binding of this tracer to other circulating T4-binding proteins (eg, TTR, TBG) (87) with almost complete sensitivity and specificity. Radiolabeled T4 or T3 bound to antibodies in serum can be precipitated by PEG, identifying the presence of anti-iodothyronine antibodies (88, 89). Similarly, radiolabeled TSH can be used to detect circulating anti-TSH antibodies and this test may be helpful in identifying weak macro-TSH–-Ig complexes (see above) which are not detected by gel filtration (85). Although many routine laboratories are now reluctant to use radiolabeled tracers and readily available, non-radiolabeled alternatives are yet to be developed, these techniques are effective screening methods.

### Genetic Testing

DNA sequencing to identify variants in genes encoding circulating TH binding proteins (*ALB*, *TBG*, *TTR*) or mediating TH action (*SLC16A2*, *SECISBP2*, *TRU-TCA1-1*, *DIO1*, *THRA*, *THRB*), enables definitive diagnosis of genetic entities and disorders.

In resource-limited settings, we suggest that using particular patterns of discordant TFTs (as illustrated in Tables 4-6) to guide and inform the selection of specific candidate genes for sequencing can be an economical approach, especially for entities (eg, FDH, transthyretinemic hyperthyroxinemia, RTHβ) where most cases are associated with a restricted repertoire of causal or pathogenic genetic variants, localizing to a few coding exons. However, as costs of this technology continue to fall, it may become cost-effective to sequence panels of genes (eg, mediating hyperthyroidism, hypothyroidism) or analyze the whole exome or genome of an individual.

**Table 6.** Causes of raised (free) T4 and (free) T3 and nonsuppressed TSH

Disorder	Transthyretinemic hyperthyroxinemia	Familial dysalbuminemic hyperthyroxinemia	Resistance to thyroid hormone β	TSHoma
Prevalence	1 in 10-10 000*^b^*	1 in 6 000*^a^*	1 in 19 000-40 000	1 in 360 000
Etiology	*TTR* mutation	*ALB* mutation	*THRB* mutation	Pituitary tumor
FT4	High	High	High	High
FT3	Normal or High	Normal or High	High	High
TSH	Normal	Normal	Normal (or Raised)	Normal (or Raised)
SHBG	Normal	Normal	Normal	High (or Normal)
Reverse T3	Raised	Normal or Raised	Raised	Raised
Clinical features	None	None	Goiter Hyperthyroid features, may be asymptomatic	Goiter Hyperthyroid features, may be asymptomatic

Abbreviations: FT3, free triiodothyronine; FT4 free thyroxine; TSHoma, thyrotropinoma.

^*a*^Prevalence varies depending on ethnicity.

^*b*^Prevalence of causal *TTR* variants in UK Biobank.

Irrespective of the approach, it is important to recognize that gene sequencing can identify variants of unknown significance. To avoid misclassification of variants and misdiagnosis as has been recently highlighted with *THRB* (90), it is important to show that the variant genotype cosegregates with abnormal thyroid function or phenotype in families. If this is not possible (eg, in sporadic cases or due to the unavailability of family members), modeling based on protein structures or even functional studies of a variant protein to establish its pathogenicity, may be required (91).

## Back to Solving the Cases

### Case 1

Further investigation of this patient showed that her circulating TT4 was raised (**249 **nmol/L [RR 69-141] or **19.34** μg/dL [RR 5.36-10.95]), but associated with a normal serum TBG (29.9 μg/mL [RR 14-31]) concentration. Then, using a validated method (86), we showed that binding of radiolabeled T4 to her serum albumin was increased, raising the possibility of an alternative binding protein abnormality—FDH. Knowing that FDH is commonly caused by a restricted repertoire of genetic variants (15), we sequenced *ALB* and identified the most common causal variant (Arg218His) in her case, as well as in her sibling with hyperthyroxinemia. Importantly, as we have documented previously with FDH (16), when her serum was tested using different immunoassay methods in current use, many recorded falsely raised FT3 as well as elevated FT4 values (Table 3). This observation, together with the familial nature of FDH, emphasizes the possibility of this entity being misdiagnosed as RTHβ or a TSH-secreting pituitary tumor, leading to unnecessary further investigation or inappropriate treatment (16).

### Case 2

After brief discontinuation of T4 to assess the HPT axis in its natural state, TFTs showed congruent TSH measurements in different assays (Perkin-Elmer DELFIA: **43.8** mU/L [RR 0.4-4]; Siemens CENTAUR **45.6** mU/L [0.35-5.5]), but discordant FT4 values using a two-step (Perkin-Elmer DELFIA: **7** pmol/L [RR 9-20] or **0.54** ng/dL [RR 0.69-1.55]) vs one-step (Siemens CENTAUR **60** pmol/L [RR 10.5-21] or **4.66** ng/dL [RR 0.81-1.63]) immunoassay methods, with confirmation of positive antithyroid peroxidase antibody status. After restarting thyroxine therapy (125** **µg daily), testing his thyroid function using different platforms showed highly concordant, near-normal TSH values, with normal FT4 measurements in two-step assays but variably discordant values in one-step methods (Table 3). As others have documented previously (25), this pattern is highly suggestive of measurement interference due to an anti-iodothyronine antibody. Ongoing titration of thyroxine replacement in this patient was guided by measurement of TSH and, if necessary, FT4 using a two-step immunoassay method.

### Case 3

When assayed in serial dilution, TSH values in maternal serum did not fall linearly (neat serum, 22.4** **mU/L; actual values on dilution 1 in 2: 23.2** **mU/L; 1 in 4: 19.3** **mU/L; 1 in 8: 12.2** **mU/L; 1 in 16: 6.4 mU/L; 1 in 32: 3.6** **mU/L), raising the possibility of assay interference. When corrected for the dilution factor, TSH concentrations appear to increase (neat: 22.4 mU/L; 1 in 2: 46.4** **mU/L; 1 in 4: 77.2** **mU/L; 1 in 8: 97.6** **mU/L; 1 in 16: 102.4** **mU/L; 1 in 32: 115.2** **mU/L)—a pattern highly suggestive of a weakly bound macro-TSH–Ig complex which dissociates on dilution. As we have documented in an unrelated case (92), gel filtration studies showed that immunoreactive TSH eluted at a higher molecular mass peak which was not present after adsorption with protein G-Sepharose, consistent with maternal serum containing a TSH–IgG complex. We surmise that transplacental passage of this antibody, causing interference in neonatal TSH screening, led to misdiagnosis of CH in both her children. Progressive disappearance of this interfering antibody from their circulation likely accounts for spontaneous reduction in (younger child) or normalization of (older sibling) TSH measurements in her children with time. A recent report documented the presence of macro-TSH in 0.43% of neonates screened (85).

### Case 4

**Figure 1.**
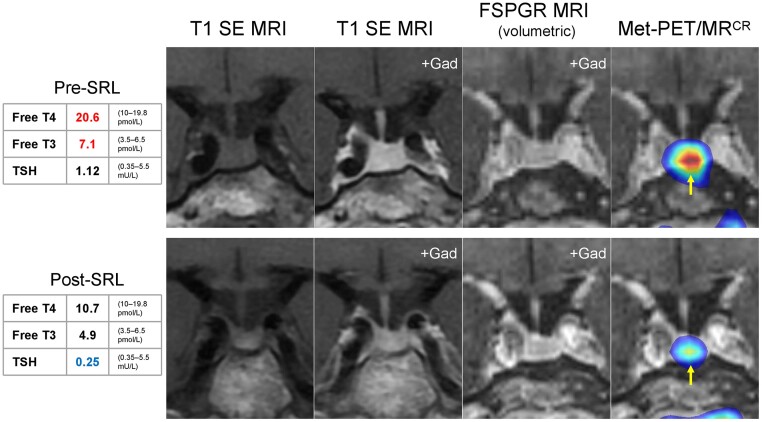
Molecular (functional) pituitary imaging in a patient with an occult microthyrotropinoma. Standard clinical MRI (coronal T1SE before and after gadolinium) at diagnosis fails to demonstrate an adenoma. Similarly, volumetric (FSPGR) sequences are unremarkable. However, molecular imaging with Met-PET coregistered with FSPGR MRI reveals intense focal radiotracer uptake just to the left of the site of insertion of the infundibulum. Following 3 months of depot SRL therapy, with resultant normalization of thyroid function tests, there is no discernible change in anatomical imaging findings; however, in marked contrast, there is dramatic diminution in radiotracer uptake, thereby revealing the location of the occult microadenoma (subsequently confirmed at transsphenoidal surgery). Abbreviations: FSPGR, fast spoiled gradient recalled echo; Gad, gadolinium; Met-PET/MR^CR^, ^11^C-methionine PET/CT coregistered with FSPGR MRI; MRI, magnetic resonance imaging; PET, positron emission tomography; SE, spin echo; SRL, somatostatin receptor ligand; T3, triiodothyronine; T4, thyroxine; TSH, thyroid-stimulating hormone.

Although administration of a short-acting SRL (octreotide) lowered circulating THs in this patient, as documented previously (93) this response does not distinguish between RTHβ and thyrotropinoma. On the other hand, the inability of long-acting (depot) SRL treatment to lower TH concentrations was highly suggestive of a diagnosis of RTHβ (93). *THRB* sequencing, identifying a heterozygous mutation (Arg438His) known to cause the disorder, confirmed this diagnosis. Thus, in patients with genuinely elevated free THs and nonsuppressed TSH, this case illustrates how coincidental anomalies on pituitary imaging can be misleading, and also the utility of dynamic investigation with administration of long-acting SRL in differential diagnosis. Indeed, as we have documented previously (94), combining long-acting SRL administration with ^11^C-methionine PET pituitary imaging (Fig. 1), enables localization of TSH-secreting pituitary microadenomas that have eluded detection with conventional magnetic resonance imaging.

## Conclusions

We have reviewed patterns of discordant thyroid function (raised [free] T4 and/or [free] T3 and nonsuppressed TSH) due to entities causing interference with measurement methods or disorders associated with genuinely altered hormone concentrations. While we have illustrated the susceptibility of most current TH immunoassay methods to measurement interference, we recognize that this is a dynamic situation. Specifically, with manufacturers continually changing the architecture and biochemical conditions of their measurement methods, it is quite possible that their susceptibility to some types of interference will be eliminated, whilst exposing vulnerability to new or different interference mechanisms.

**Figure 2.**
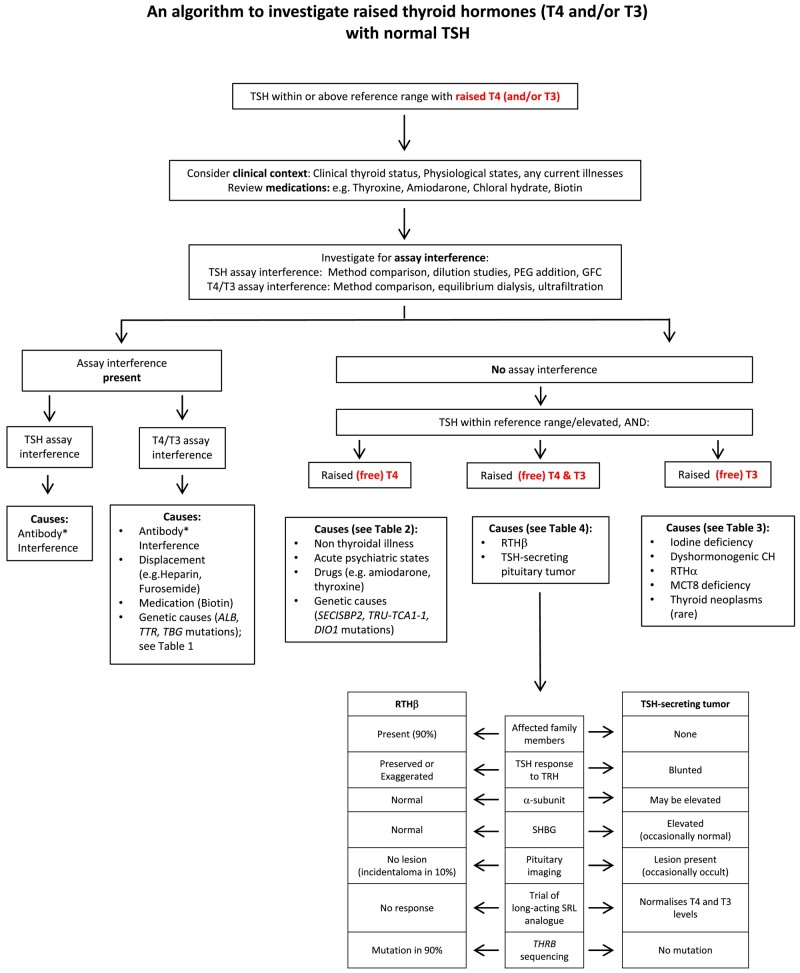
Antibody interference in free T4/T3 measurement can be due to anti-iodothyronine or antireagent antibodies, and in TSH measurement due to anti-TSH (macroTSH) or antireagent antibodies Abbreviations: CH, congenital hypothyroidism; GFC, gel filtration chromatography; PEG, polyethylene glycol; RTHα, resistance to thyroid hormone α; RTHβ, resistance to thyroid hormone β; SHBG, sex hormone–binding globulin; SRL, somatostatin receptor ligand.

In our overall approach to evaluation of raised THs and nonsuppressed TSH, we have outlined an algorithm for differential diagnosis (Fig. 2), which we hope is both economical and applicable in resource-limited settings.

Finally, with one review suggesting that over 50% of cases with discordant thyroid function were associated with misdiagnosis, inappropriate investigation and management (24), the potential for preventing unnecessary costs and adverse health outcomes via a structured approach to this biochemical entity cannot be underestimated.

## Data Availability

Data sharing is not applicable to this article as no datasets were generated or analyzed during the current study.

## Abbreviations

CH: congenital hypothyroidism
FDH: familial dysalbuminemic hyperthyroxinemia
FT3: free triiodothyronine
FT4: free thyroxine
HPT: hypothalamic–pituitary–thyroid
MCT8: monocarboxylate transporter 8
PEG: polyethylene glycol
SHBG: sex hormone–binding globulin
SRL: somatostatin receptor ligand
T3: triiodothyronine
T4: thyroxine
TBG: thyroxine binding globulin
TFT: thyroid function test
TH: thyroid hormone
TSH: thyrotropin
TSHoma: thyrotropinoma
TT4: total T4
TTR: transthyretin
